# Natural Polysaccharide Nanomaterials: An Overview of Their Immunological Properties

**DOI:** 10.3390/ijms20205092

**Published:** 2019-10-14

**Authors:** Fernando G. Torres, Omar P. Troncoso, Anissa Pisani, Francesca Gatto, Giuseppe Bardi

**Affiliations:** 1 Department of Mechanical Engineering, Pontificia Universidad Catolica del Peru, Av. Universitaria 1801, Lima Lima 32, Peru; fgtorres@pucp.pe (F.G.T.); troncoso.op@pucp.pe (O.P.T.); 2 Nanobiointeractions & Nanodiagnostics, Istituto Italiano di Tecnologia, Via Morego 30, 16163 Genova, Italy; anissa.pisani@iit.it; 3 Department of Chemistry and Industrial Chemistry, University of Genova, Via Dodecaneso 31, 16146 Genova, Italy; 4 Drug Discovery and Development Department, Istituto Italiano di Tecnologia, Via Morego, 30, 16163 Genova, Italy; francesca.gatto@iit.it

**Keywords:** Polysaccharides, Immune System, Nanomaterials

## Abstract

Natural occurring polymers, or biopolymers, represent a huge part of our planet biomass. They are formed by long chains of monomers of the same type or a combination of different ones. Polysaccharides are biopolymers characterized by complex secondary structures performing several roles in plants, animals, and microorganisms. Because of their versatility and biodegradability, some of them are extensively used for packaging, food, pharmaceutical, and biomedical industries as sustainable and renewable materials. In the recent years, their manipulation at the nanometric scale enormously increased the range of potential applications, boosting an interdisciplinary research attempt to exploit all the potential advantages of nanostructured polysaccharides. Biomedical investigation mainly focused on nano-objects aimed at drug delivery, tissue repair, and vaccine adjuvants. The achievement of all these applications requires the deep knowledge of polysaccharide nanomaterials’ interactions with the immune system, which orchestrates the biological response to any foreign substance entering the body. In the present manuscript we focused on natural polysaccharides of high commercial importance, namely, starch, cellulose, chitin, and its deacetylated form chitosan, as well as the seaweed-derived carrageenan and alginate. We reviewed the available information on their biocompatibility, highlighting the importance of their physicochemical feature at the nanoscale for the modulation of the immune system.

## 1. Introduction

Living organisms synthetize polymers during their entire life cycle. Naturally occurring polymers, or biopolymers, can be found in plants, animals, bacteria, and fungi. They display a huge diversity of monomers and secondary structures. The main groups of biopolymers include proteins, nucleic acids, polyhydroxyalkanoates, polyphenols, and polysaccharides. Some of these polysaccharides such as starch, cellulose, carrageenan, or alginate are industrially extracted on large scale for applications as stabilizers, excipients, thickeners, and gelling agents. Others are low-value waste products like lignin, which is a by-product from the Kraft pulping process. Chemically and structurally diverse biopolymers found in natural systems are widely used for packaging, food, pharmaceutical, and biomedical industries as sustainable, renewable, biodegradable, and non-toxic agents. The molecular structure of biopolymers is highly hierarchized [1] and their features (i.e., chain lengths, monomer sequence, etc.) are optimized for specific biological functions. Furthermore, all the natural polymers are “adaptable materials”, as their configurations are constantly manipulated by enzymes during the different life cycle stages in response to environmental changes [2].

Nucleic acids, proteins, and polysaccharides display properties that cannot be attributed to the individual building blocks (monomers) and are associated with their complex secondary and tertiary organizations [3,4,5]. Remarkably, some of these features can be exploited for biomedical applications. Among the same class of polymer, a different quality may depend on the biological source and methods of extraction. For example, morphology, crystallinity, gelatinization temperature, and other characteristics of starch depend on the botanical source (potato, corn, etc.) and methodology used for its purification. This knowledge is crucial for the synthesis of polysaccharide nanomaterials, for example, for the preparation of polysaccharide nanoparticles (NPs) with a defined size, shape, and stiffness.

Natural polysaccharides can be combined with other materials to form novel composites showing improved properties. Most of these complexes are usually polymers reinforced with a second micro-sized material, whereas nano-sized (1–100 nm) structures are complexed in “nanocomposites”. According to the micromechanics laws, the effective properties of “microcomposites” are mainly a function of the polymer properties and the volume fraction of their constituents, but are independent of the reinforcements’ size. Differently, when scale effects take place for nanocomposites, some forces are irrelevant while other forces become considerable [6,7,8,9]. For instance, gravitational and inertial forces are negligible while van der Waals, electrostatic, and adhesive forces are significant. In addition, in colloidal suspensions of NPs for drug delivery or diagnostics, the Brownian motion of NPs influences their nano-biointeractions. Nanocomposite materials for biomedical applications can be tailored to improve their interaction with biological systems, leading to the design of truly effective and biocompatible biomedical devices.

In view of potential biomedical applications, the biocompatibility of natural occurring materials represents the first and most important feature to be considered. Biocompatibility can be defined as the ability to produce an appropriate host reaction without eliciting adverse effects [10]. Like any foreign substance that enters the body, these materials induce biological responses aimed at their clearance. If these responses are exaggerated and uncontrolled, they may induce wide-ranging and long-term toxicity involving the immune response against foreign materials as a crucial step to distinguish between their biocompatibility or toxicity.

The immune system (IS) is an extremely complex and coordinated network constituted of several cell types and cellular mediators that allow discrimination between self and non-self molecules, whose modulation rules the induction, enhancement or suppression of the response. In vertebrates, the IS is functionally divided in two different sub-systems: innate and adaptive, which are evolutionary superimposed. Both of these sub-systems cooperate in a joined manner to create an appropriate and balanced response versus external and internal hazards [11].

The innate immunity results in an immediate and non-specific defense mediated by phagocytic cells and molecules that regulate systemic or local events including inflammation, coagulation, and the complement cascade activation. The complement comprises a group of blood proteins that causes target opsonization, phagocyte recruitment, and localized inflammation following enzymatic cascade activation [12].

Phagocytes, such as monocytes/macrophages or neutrophils, operate encapsulating and destroying foreign bodies through specific enzymes and controlled reactive oxygen species (ROS) production. As sentinels of our bodies, monocytes detect environmental signals, driven by specific chemo-attractant molecules (chemokines) released by tissue cells. Pro-inflammatory signals induce monocytes to penetrate tissues and differentiate into macrophages, phagocyting pathogens, and damaged cells. The engagement of specific pattern recognition receptors (PRRs) expressed on the cell membrane with conserved exogenous structure, called pathogen-associated molecular patterns (PAMPs), or with endogenous molecules released following an injury (damage-associated molecular patterns (DAMPs)) stimulates phagocytosis [13]. Neutrophil and macrophage activation can be achieved via different kinds of PRRs, such as Toll-like receptors (TLRs), C-type lectin receptors (CLRs), Retinoic acid-inducible gene (RIG)-I-like receptors (RLRs), and NOD-like receptors (NLRs). These receptors can work separately or forming signaling clusters. With the exception of some NLRs, the activation of PRRs upregulates the transcription of genes involved in the inflammatory response, mainly including the transcription factor NF-κB. The expression pattern of the inducible genes differs among the various PRRs and includes pro-inflammatory cytokines, type I interferons (IFN), chemokines, and antimicrobial proteins, as well as proteins that modulate PRR signaling. Specific cytokines, like interleukin (IL)-1β, IL-6 and tumor necrosis factor (TNF), orchestrate the pro-inflammatory response with pleiotropic effects [14].

In addition to phagocytosis, activated macrophages regulate the growth and the differentiation of other cells through the release of molecular mediators linking the innate to the adaptive immunity and tailoring the response towards specific threats [15]. Actually, the acquired immunity provides exclusive and long-term defense by means of immunological memory. Selected subsets of previously primed B and T lymphocytes maintain the ability to rapidly respond to a second pathogen encounter [16]. Lymphocytes’ memory can be also artificially created by vaccination with antigens that mimic the presence of a specific pathogen. Several vaccines, or immune-modulatory therapeutics against microbial infections are created by isolating the polysaccharide bacterial antigen and conjugating it to an immunogenic carrier [17]. Different immunomodulating biomaterials have been proposed for novel formulations of nanovaccines. Among them, carbohydrate-based materials are currently attracting great attention as immune stimulators [18]. Polysaccharides may have different immune effects, primarily the activation and/or the enhancement of macrophage response by increasing ROS production and cytokines secretion [19]. Due to these intrinsic features, several studies have investigated the potential employment of polysaccharides as non-toxic vaccine adjuvants to enhance the immune response towards several antigens. In particular, polysaccharides can behave as molecular adjuvants by activating cellular PRRs [20].

In this review we will consider the immune interactions of common natural polysaccharides (i.e., starch, cellulose, chitin, chitosan, carrageenan, and alginate) and their nanocomposites such as NPs, nanofibers (NFs), nanowhiskers (NWs), or nanocrystals (NCs).

## 2. Natural Polysaccharides and Polysaccharide Nanomaterials

Polysaccharides are macromolecules composed of sugar units linked by glyosidic bonds. They represent, by far, the most abundant natural polymers on earth [21,22]. The amount of polysaccharide produced by plants per year exceeds by several orders of magnitude the synthetic production by the chemical industry [23]. Natural polysaccharides (Table 1) are synthetized to fulfill many different functions, such as energy storage in plants (i.e., starch), structural support of vegetal cells (i.e., cellulose), gelling agents forming the intercellular matrix and containing several ions such as sodium, calcium and magnesium (i.e., alginate in the brown algae). Some of them like cellulose, starch, chitin, and polysaccharides from seaweeds are commercially important in several markets, ranging from paper production to food industry products. We will restrict our description to those polysaccharides and their interactions with the immune system.

### 2.1. Starch

**Figure 1:**
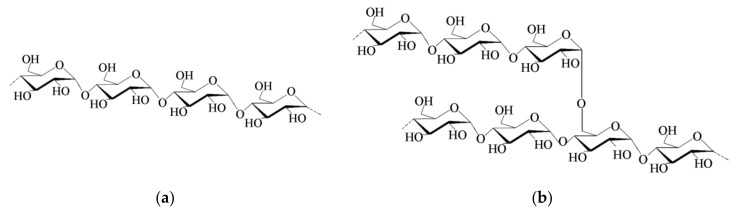
Schematic representation of amylose (**a**) and amylopectin (**b**) macromolecules.

Starch is produced by plant in the form of granules mainly containing two biopolymers: amylose and amylopectin (Figure 1). The amount of amylose is around 20–30% while amylopectin is around 70–80%. Other minor constituents of starch granules are lipids, proteins, and minerals. The amounts of all these constituents vary with the botanical origin [24]. The botanical sources also determine the main characteristics of starch granules such as size and shape. Amylose (Figure 1a) is a linear macromolecule made up of α-(1-4)-d linked polyglucan in a helix conformation [25]. Amylopectin (Figure 1b) has a branched configuration with α-(1-4)-d glucopyranose backbone and 5–6% of α-(1-6)-branch linkages [26]. Amylopectin chains form double-helices, which crystalize and contribute to the semi-crystalline nature of the starch granules [27]. Under the microscope, starch granules exhibit a Maltese cross when observed under polarized light [28]. This is due to the densely packed starch granules with semi-crystalline structures, in which the crystallinity varies from 15% to 45%. The birefringence is due to the radial orientation of the principle axis of the crystallites. Starch granules are insoluble in water and alcohol but can be dissolved in hot water during a process known as gelatinization. During gelatinization, the starch granules are irreversibly destroyed releasing the amylose and amylopectin biopolymers. The temperature at which gelatinization process takes place is around 70 °C for most starch granules, undergoing an irreversible transition in which the molecular structure of amylose and amylopectin is disrupted. The amorphous regions swell by the absorption of water (or other plasticizer) and the crystalline regions are destroyed [29,30]. As result, starch can be processed as a thermoplastic and is referred to as thermoplastic starch (TPS). TPS can be further treated using standard plastic processing techniques such as extrusion and injection molding in which temperature, pressure and the addition of a plasticizer (e.g., water, glycerol, etc.) trigger gelatinization and transform the starch granules into a homogeneous material. Starch is non-toxic, renewable, and biodegradable. In fact, it is employed as an additive in the adhesive, paper, and clothing industries and, as raw material, for the production of biodegradable plastics for the packaging industry.

Starch can be used to produce films with nanostructured surfaces, NPs, as well as hetero-materials nanocomposites. The acid hydrolysis can remove the amorphous domains and break down starch granules to NPs from few (5–30 nm) to several hundred of nanometers (100–300 nm) [31,32,33,34,35,36,37,38,39].

Besides acid hydrolysis, high-pressure homogenization, sonication, and microemulsion based techniques, as well as combinations of them, are employed to produce starch NPs [40,41]. For instance, hydrolysis followed by emulsification. The processing method crucially determines the characteristics of the obtained NPs. We studied [42,43,44] how NP properties can be tuned by using different botanical sources (different varieties of potato) and processing protocols. Our results demonstrated, for example, that NPs formed by acid hydrolysis are size-dependent on the botanical origin, whereas NPs formed by nanoprecipitation are size-independent of their biological source.

Biodegradable all-starch nanocomposite films can be also obtained by casting. Cassava, waxy maize, potato, and rice have been reported as suitable sources to prepare all-starch nanocomposites [36,45,46,47,48,49]. Our group investigated the effect of using a specific starch source to prepare the matrix combining NPs from three different botanic species [49]. The results showed that the mechanical properties of the nanocomposite films vary depending on both the amount of reinforcement present and the source of the starch. So, processing routes, vegetable starch varieties, or the appropriate combination of them must be carefully selected to tailor the final NP features (size, amylose content, and crystallinity percentage) for the intended application.

**Figure 2:**
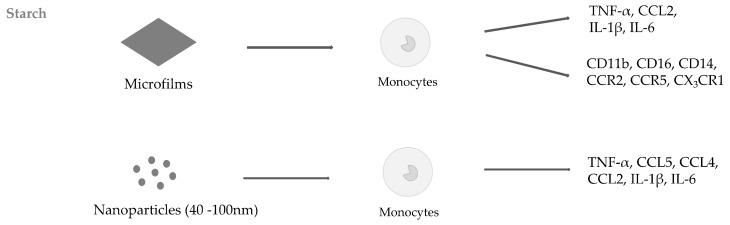
Representation of cytokine release and immune receptor expression induced by starch microfilms and polysaccharide nanoparticles (NPs).

Several studies suggest that starch-based nano and micro-materials can be used for biomedical applications, like tissue engineering, cell growth substrates, wound healing patches, and drug delivery systems [42,50,51]. Although starch nanomaterials (NMs) are generally not toxic, their properties can induce immune responses. In one of our studies investigating the immunological properties of microfilms prepared with starch extracted from six different types of Andean potatoes, we demonstrated that they differed in the ability to induce immune activation of THP-1 monocytes [32]. We showed that all the six tested starches did not impact the cell viability but differently induced the expression of pro-inflammatory cytokines, such as TNF-α, IL-1β, IL-6, and CCL2. The six microfilms also induced different chemokine and immune-related receptor expression (i.e., CD11b, CD16, CCR2, CCR5, CD14, and CX_3_CR1) on THP-1 membrane. The nanometric differences of the film surfaces and the dissimilar stiffness of the six materials did not correlate with their immunogenic activity. We hypothesized that the different Andean-native potatoes’ starch films had specific ability to interact with the membrane of immune cells, plausibly due to the diverse spatial localization of amylose and amylopectin of the starches. We obtained similar results using NPs (from 40 to 100 nm) prepared with starches extracted from two different potatoes, *Solanum tuberosum* (common name “Negrita”, NEG) and *Solanum goniocalyx* (common name “Peruanita”, PER) [51]. In particular, we showed that only the administration of NEG NPs to the cells induced a significant release of IL-6, IL-1β, TNF-α, CCL2, CCL4, and CCL5, while PER NPs did not induce any significant cytokine release in THP-1 monocytes. Figure 2 collectively summarizes the described observations using Andean-native potato starches.

Biocompatibility studies of starch-based polymers and composites, both in vitro and in vivo, has been presented by Marques et al. [52,53]. Initially they performed cytotoxicity and cell adhesion tests of two blends of corn starch, namely, starch/ethylene vinyl alcohol (SEVA-C) and starch/cellulose acetate (SCA), as well as their respective composites with hydroxyapatite (HA). They found that both types of starch-based polymers and composites exhibit a good cytocompatibility and adhesion on L929 mouse fibroblasts, although cells preferentially adhered to SCA surface. The authors also compared the ability of the starch/polycaprolactone (SPCL) and its HA composite with poly-l-lactide (PLLA) to induce cytokine production by mononuclear cells in vitro [54]. The results demonstrated no expression of IL-2 or IFN-γ induced by any of the materials within 14 days. Conversely, the production of both IL-6 and TNF-α was high in all the tested conditions. IL-1β was produced in very low amounts and IL-4 was expressed without any difference within the group of materials. In particular, the authors showed that the more hydrophilic surface of SCA induced higher TNF-α and IL-1β expression, whereas SPCL with the most hydrophobic surface stimulated the highest secretion of IL-6. The presence of HA resulted in a significant reduction of those inflammatory cytokines, especially in the case of SCA. Nevertheless, the proinflammatory cytokine production by all the starch composites was lower compared to PLLA. The authors also investigated the same materials in vivo by subcutaneous implants using Wistar rats [53]. Immunohistochemical evaluation of the tissues surrounding the graft revealed that after retrieval, only mild inflammatory reaction was present. However, among the tested materials, the composite SPCL and SCA stimulated the most evident responses. These studies highlight the immunological importance of the polysaccharide interactions with the cell surface.

Because of the immune-stimulating properties of starch-based materials some researchers investigated their possible role as vaccine adjuvant. Mendieta et al. explored the potential of starch microparticles (µP) to enhance Mycobacterium tuberculosis heat shock protein alpha-crystallin specific immune response [55]. They immobilized the heat shock protein on the microparticles and nasally administered the µP-antigen system or the µPs alone to BALB/c mice with progressive pulmonary tuberculosis. The results showed that the presence of either starch µPs with the immobilized antigen or starch µPs alone boosted BCG vaccination (in BCG-vaccinated mice without any extra adjuvant) inducing a significant reduction of bacterial presence in the mice lungs. In particular, since no difference was observed in pulmonary bacillary burdens between the reinforced groups the authors suggested that the effect was primarily caused by the starch.

Although diverse immune reactions have been associated with the starch administration in specific biomedical models, precise mechanisms regarding immune interactions are still poorly understood and require further investigation.

### 2.2. Cellulose

Cellulose is a highly abundant natural polysaccharide and the main structural material of plant cell walls making up about half of the biomass of photosynthetic organisms [56]. It is exhaustively used in the paper industry; however, the abundance of this renewable resource makes cellulose a good candidate for the production of sustainable biopolymer-based materials.

**Figure 3:**
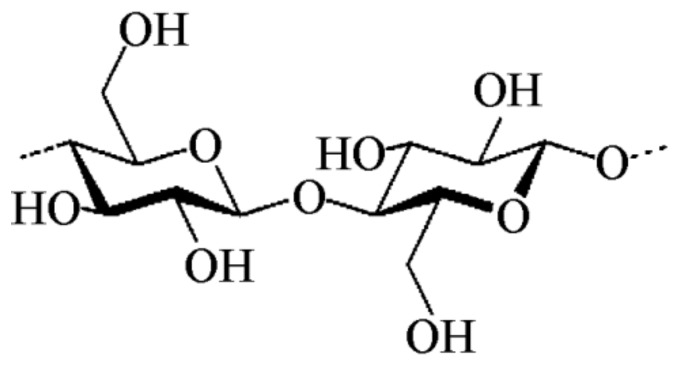
Schematic representation of a cellulose macromolecule.

At the molecular level, cellulose is a linear homopolymer of β-(1→4)-linked d-glucopyranosyl units with a degree of polymerization of around 10,000–15,000 [57] (Figure 3). At a nano-level, cellulose molecules form elementary fibrils. It has been reported that during cellulose biosynthesis, van der Waals and intermolecular hydrogen bonds between hydroxyl groups and oxygens of adjacent molecules promote parallel stacking of multiple cellulose chains forming elementary fibrils [58,59]. The fibrils have a square cross-section of 3–5 nm in size composed by around 36 individual cellulose molecules and further assemble into microfibrils with a cross-section of 20 nm × 8 nm [58,60].

Within the elementary fibrils ordered (crystalline) and disordered (amorphous) regions can be found. The crystallinity of cellulose depends on the botanical source. For example, cellulose from cotton is more crystalline (~80% crystallinity) compared to wood cellulose (~50% crystallinity) [61]. Four different polymorphs of crystalline cellulose exist, namely, cellulose I, II, III, and IV. Cellulose I is naturally produced by living organisms like plants, algae, and bacteria as two subtypes: Iα and Iβ. Cellulose Iα has a triclinic crystalline structure and is found mostly in algae and bacteria while cellulose Iβ has a monoclinic structure and is present in plants and tunicates (the only known animal able to synthetize cellulose). The high crystallinity of cellulose is a key property for the development of new cellulose-based nanocomposites. When the amorphous regions of cellulose are removed, the crystalline regions form cellulose nano-whiskers or cellulose nano-fibers. Another form of nano-cellulose is synthetized by bacteria as fibers with ~100 nm in diameter. These nano-sized celluloses have been used to produce nanocomposites with excellent mechanical properties for a variety of applications, including tissue engineering, wound healing, and cell growth [62].

Native cellulose fibers can be disintegrated into nanoscale morphologies through different processing routes. The resulting structures are usually classified as cellulose micro or nanofibers (NFs), NWs, and NPs. Table 2 shows the diameter (D), length (L), and aspect ratio (L/D) of the cited nano-objects. The most used processing techniques include mechanical and chemical treatments. The mechanical processes reported comprise mechanical-grinding, cryo-crushing, and high-pressure homogenization [63]. Any of these mechanical processes allows the generation of cellulose microfibers, whereas in order to obtain NFs, NWs, or NPs from plant cellulose, a combination of chemical and mechanical treatment is usually needed. The chemical reactions include enzymes-based treatments, acid hydrolysis (sulfuric, hydrochloric, or phosphoric acids), 2,2,6,6-tetramethylpiperidine-1-oxyl-mediated oxidation (TEMPO-mediated oxidation), carboxymethylation, and acetylation [64]. NFs produced using bacterial cellulose can be advantageous, since bacteria like *Gluconacetobacter* synthetizes pure cellulose in the form of NFs (~100 nm in diameter) not requiring further treatments. All-cellulose nanocomposites are commonly made by dissolving a portion of cellulose in a solvent to obtain the matrix. Cellulose is then regenerated in the presence of undissolved whiskers forming transparent films with high mechanical properties [65,66].

Cellulose NFs can also be used to prepare polycomponent nanocomposites. For instance, Grande et al. [67] reported a bottom-up processing methodology to prepare nanocomposites of starch and bacterial cellulose. Cellulose NFs were synthetized in culture medium previously enriched with starch granules. This combination produced starch-bacterial cellulose nano-films. A similar route was reported to create hydroxyapatite-bacterial cellulose nano-structures for biomedical applications [68].

Even though cellulose-based NMs are considered biocompatible materials, some studies reported their harmfulness and induced immune reactions. Several parameters contribute to the cell viability outcome such as NM concentration, shape, surface area, and charge, as well as the source of cellulose, NM preparation, and their degree of agglomeration. Actually, the biological environment and the chosen testing assays only provide limited information regarding nanocellulose biocompatibility [76,77,78]. For example, Dong et al. tested the cytotoxicity of cellulose nanowhiskers in nine different cell lines [79]. Cell viability was evaluated using NM concentration up to 50 µg/mL for 48 h both by 3-(4,5-dimethylthiazol-2-yl)-2,5-diphenyltetrazoliumbromide) (MTT) and Lactate Dehydrogenase (LDH) assay. No cytotoxic effects of cellulose NWs were reported. Interestingly, Catalán and colleagues [80] observed that 100 µg/mL cellulose NWs (135 nm length, 7.3 width) as well as bigger 50 µm particles (microcrystalline cellulose (MCC)) caused 55% human bronchial epithelial BEAS 2B cell death within 48 h. Moreover, 300 µg/mL MCC induced the release of the pro-inflammatory cytokine TNF-α and enhanced LPS-induced IL-1β release in human monocyte-derived macrophages. The nanocrystal fibers at the same concentration did not produce proinflammatory cytokine release. No inflammatory reactions nor cytotoxicity of cellulose nanofibrils (CNFs) in mouse and human macrophages have also been reported by Vartiainen et al. [81]. VTT Technical Research Centre in Finland proposed a systemic study of CNFs evaluating in vitro cytotoxicity, genotoxicity, immunotoxicity, neurotoxicity, and pharyngeal aspiration study on mice [82]. The results revealed low cytotoxicity and no DNA or chromosome damage by nano-fibrils. However, pulmonary inflammation in mouse experiment was detected. The authors suggested that it was probably induced by particulate/bacteria contamination of the cellulose preparation. Uncontrolled contamination with endotoxins or preparation process by-products represents a big problem for the interpretation of several data collected in many experimental conditions.

Other researchers showed that needle-like cellulose fibrils, prepared by acid hydrolysis of cotton cellulose fibers decreased the viability of bovine fibroblasts in vitro at high concentrations (2 mg–5 mg/mL) [83]. Furthermore, these fibrils upregulated the expression of stress and apoptosis related molecules. However, their concentrations up to 100 µg/mL did not influence cellular viability.

**Figure 4:**
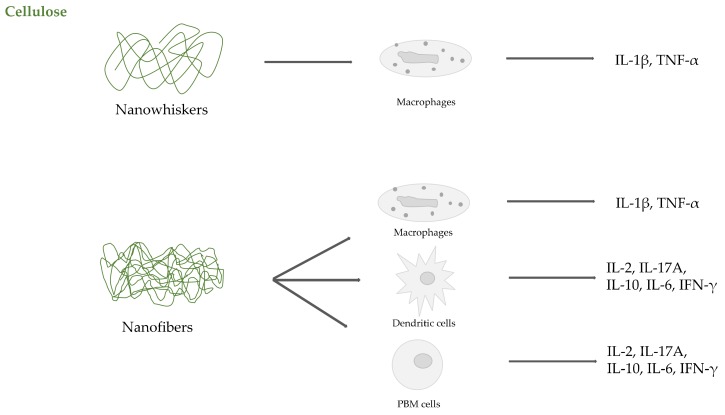
Cytokine release induced by cellulose nanostructures.

Studies on CNFs have been also performed with wood-derived cellulose by Čolić et al. [84]. The authors cultured L929 mouse fibroblast, rat thymocytes, and human Peripheral Blood Mononuclear Cells (PBMCs) in the presence of six different concentrations of wood-based CNFs, ranging from 31.25 µg/mL to 1 mg/mL. They observed that CNFs, with diameters of 10–70 nm and lengths up to 30 microns, did not induce cytotoxicity or oxidative stress in the L929 cells, nor produced necrotic or apoptotic cell death of thymocytes and PBMCs. Higher concentrations (250 µg/mL–1 mg/mL) slightly inhibited the metabolic activities of L929 cells as a consequence of inhibited proliferation. The same concentrations of CNFs suppressed the proliferation of PBMCs in response to the T-cell mitogen PHA, as well as the down-regulation of IL-2 and IFN-γ production. Only at the highest concentration, CNFs were able to inhibit IL-17A, contemporaneously increasing the anti-inflammatory IL-10 and the pro-inflammatory IL-6. The secretion of other pro-inflammatory cytokines, like IL-1β, TNF-α, as well as the Th2-produced cytokine IL-4, remained unaltered. Similar results by the same authors obtained in a different study using dendritic cells [85] suggest that CNFs are not significantly immunogenic, although they interfere with monocyte-derived dendritic cell differentiation in vitro. Figure 4 summarizes the main evidence regarding cytokine release induced by cellulose nanostructures.

In addition to shape and concentration of cellulose-based NMs, charged groups on their surface can induce different toxic and immunological effects. Lopes et al. reported that unmodified CNFs and modified CNFs gels with carboxymethyl and hydroxypropyltrimethylammonium surface groups did not cause cytotoxicity in human dermal fibroblasts, lung, and macrophage cells. ROS production by THP-1 macrophages was also not detected [86]. However, unmodified CNFs induced an increase in TNF-α and IL1-β levels in THP-1 macrophages whereas the same effect was absent when the cells were treated with modified CNFs. A similar study showed that CNFs modified with the crosslinking agent polyethyleneimine and the surfactant cetyl trimethylammonium bromide caused a significant reduction in fibroblast cell viability and proliferation compared to the pure CNFs [87].

Nanocellulose produced by bacteria, known as bacterial cellulose (BC) is considered to be one of the most biocompatible materials and is employed in food industry, textiles, and medicine production. Several studies demonstrated no cytotoxic effects induced by BC on osteoblast cells, endothelial cells, and during mouse feeding experiments [88,89,90]. Furthermore, an in vivo study of subcutaneous BC implantation in rats reported that after 12 weeks, no fibrotic capsule or giant cells were detectable by microscopy, indicating no foreign body reaction. Macroscopically, no redness, swelling, or exudate developed around the implantation sites, demonstrating the absence of immune responses [91].

On the whole, there is no evidence of serious influence or damage elicited by nanocellulose at cellular, tissue or organ level. However, nanocellulose long-term in vivo toxicity and interactions with immune system still need deeper research before being translated in clinical tools, since their very complex structural combinations and physicochemical parameters may result in unpredictable immune reactions.

### 2.3. Chitin and Chitosan

Chitin is another very abundant natural polymer. It is part of the exoskeleton of insects, crustaceans, and other organisms like spore of fungi [92]. Commercially, crab and shrimp waste from the seafood industry are the most important source of chitin [93]. According to Merzendorfer [94] more than 10,000 tons could be available every year from shellfish waste.

**Figure 5:**
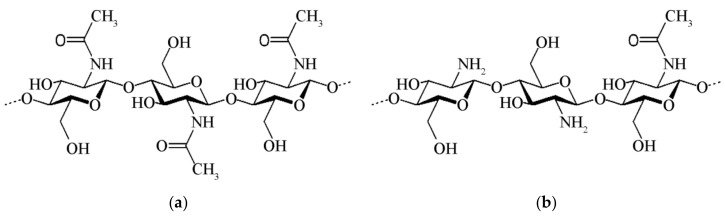
Schematic representation of (**a**) chitin and (**b**) chitosan macromolecules.

Chitin is a linear biopolymer of 2-acetamido-2-deoxy-d-glucopyranose linked together by β(1→4) glycosidic bond (Figure 5a). Chitin and cellulose show many similarities. Their macromolecules are almost identical except for the hydroxyl groups of the cellulose chain, which are substituted with acetamido groups in the chitin [92]. The hierarchical organization of chitin is also arranged in highly crystalline microfibrils formed by NFs of about 2−5 nm diameter and about 300 nm in length embedded in a protein matrix [95,96]. Like cellulose, chitin can be considered a “structural biopolymer” strengthening the exoskeleton of arthropods. The most common polymorphic forms of chitin are α-chitin and β-chitin [97]. α-chitin is found in the shells of crustaceans, exoskeleton of insects, and the fungal cell wall [22,98,99], whereas β-chitin is less common and present in squid pens and some mollusk shells [100,101]. The main difference between α- and β- polymorphs is the alignment of the polymer chains in the chitin crystals. In β-chitin all polymeric chains are aligned in parallel mode, while in α-chitin are packed in an antiparallel manner [102].

As previously discussed, cellulose and chitin structures are similar. The methods reported to obtain chitin nano-objects are similar to some described for cellulose. However, unlike cellulose, only two types of chitin nano-objects are reported, specifically NWs and NFs. NWs have a diameter of 6 to 60 nm and a length of 100–800 nm, whereas NFs have a diameter that ranges from 10 to 100 nm and a length of around 1000 nm. Chitin NWs are usually obtained by acid hydrolysis, removing the amorphous regions. Disordered and low lateral ordered crystalline defects of chitin are removed while crystalline residues remain intact [103] with a resulting crystallinity of 60–90% [104]. Other chemical treatments reported to prepare chitin NWs are 2,6,6-tetramethylpiperidinooxy (TEMPO) mediated oxidation and partial deacetylation. In contrast, mechanical procedures like grinding and homogenization, together with ultrasonic techniques, are used to obtain chitin NFs.

Chitin is hydrophobic, insoluble in water and most organic solvents. This makes chitin difficult to process and limits its applications. Nevertheless, chitosan, the deacetylated derivative of chitin, is soluble in weak acidic solutions such as formic, acetic, oxalic, and lactic acids (Figure 5b).

The degree of acetylation (DA), which is calculated as the proportion of *N*-acetyl-d-glucosamine units with respect to the total number of units, is a key parameter and determines the solubility of these two biopolymers in acid solutions. Chitosan DA is generally below 50% while the DA of chitin is typically around 90% [105]. Chitin and chitosan are used in different applications including waste water treatment [106,107], packaging materials [108], food additives [109], seed coating [110], and cosmetics [111]. Some of the particular properties of chitin and chitosan can be exploited for biomedical applications. In particular, the reduced DA of chitosan increases the range of its potential uses. Since chitosan is insoluble in neutral and alkaline environments but soluble in an acidic environment, it is exploited to develop responsive controlled drug delivery systems [112].

Chitosan reinforced with chitin whiskers is an example of chitin-based nanocomposite. Incorporation of chitin whiskers into a chitosan matrix improves the tensile strength and the water absorption resistance [113]. The interaction of the chitosan matrix with chitin whiskers can be further improved using a crosslinker such as glutaraldehyde and polydimethylsiloxane [114,115]. As versatile material, chitosan can also be reinforced with other natural polymers. For instance, Corsello et al. [116] prepared chitosan-cellulose whiskers films by casting of dispersions. The composition of the films varied from 1 to 10 wt.% of cellulose whiskers. FTIR tests revealed the presence of weak interactions between chitosan and cellulose. Celebi and Kurt [117] also prepared chitosan films reinforced with cellulose whiskers by casting. By comparing mechanical stirring-ultrasonication and microfluidization they studied how the dispersion method of cellulose whiskers in the chitosan matrix determines the interaction between the two polymers. They found that microfluidization promotes the formation of hydrogen bonds between chitosan and cellulose whiskers. This high interaction increased the crystallinity of these nanocomposites.

Chitin bio- and immune-compatibility has been proved in several experimental settings [118,119]. Among them, Sum Chow et al. reported that porous chitin matrices were non-cytotoxic for mouse and human fibroblast cell lines [120]. Another study by Dev et al. demonstrated by MTT assay also showed that carboxymethyl chitin NPs were non-toxic for L929 mouse cells [121]. Nevertheless, chitin can be sensed by the innate immune system showing modulation of the adaptive response. As for other polysaccharide NMs, chitin physical features including size, shape, source, and purification method affect immune recognition, cytokine profile, and inflammatory cell recruitment [122,123]. For example, pretreatment of mice with purified chitin particles from *Candida albicans* enhances their survival after experimental infection with the same pathogen [124]. The authors suggest that the candidacidal activity of peritoneal macrophages was boosted by chitin-induced ROS generation. On the other hand, a different study reported that ultrapure chitin purified from *C. albicans* failed to induce significant immune responses when incubated with human PBMCs [122]. A critical role of the particle size might help to interpret the data, since Da Silva et al. demonstrated the relevance of chitin micro-fragment size to induce acute proinflammatory effects in murine macrophages [125]. The authors found that large chitin fragments (70–100 μm) were inert, while both intermediate-sized chitin (40–70 μm) and small chitin (2–10 μm) stimulated TNF-α expression but only small chitin induced IL-10 production. In particular, the effects of 40–70 μm chitin were mediated by pathways involving TLR-2, dectin-1 and NF-κB. Small chitin fragments activated TLR-2 dependent and independent pathways as well as the dectin-1 dependent pathway involving the mannose receptor and spleen tyrosine kinase. Chitin can be sensed by the immune system as PAMP through specific membrane-bound receptors, triggering various molecular signaling cascades that can alter cytokine release and differentiate the cells into distinct phenotypes (i.e., from immature to mature dendritic cell; from monocyte to macrophage; etc.). As reported by Locksley and colleagues, chitin exposure increased the expression of CCL2, IL-25, IL-33, and TLSP also by lung epithelial cells and induced type 2 innate lymphoid cells (ILC2) to secrete IL-5 and IL-13 cytokines [126]. Furthermore, chitin can induce the accumulation of IL-4-expressing innate immune cells in mice tissues as shown by Reese et al. Specifically, chitin mediated alternative macrophage activation suggesting that these cells might represent sensors for chitin in tissues [127]. Likewise, chitin-mediated enhancement of T cell functions, NK cell activity, and IFN-γ production by NK cells has been proved [118]. A different in vivo study reported positive effects in the control of intestinal inflammation due to chitin microparticles (CMPs) [128]. In the experiment CMPs (1.5 mg/day/orally) or Phosphate buffer saline (PBS) were administered to acute and chronic colitis models (C57Bl/6 WT and C57Bl/6 TCRαKO mice) every 3 days for 6 weeks. The authors showed that mesenteric lymph node (MLN)-derived CD4+ T cells from CMP-treated TCRαKO mice co-cultured with dendritic cells (DCs) produced a sevenfold higher amount of IFN-γ in the presence of CMPs in comparison to PBS-treated control mice. Furthermore, immunomodulatory effects have been demonstrated by the production of IL-10 in the noninflamed colon.

The chitin derivative chitosan is also considered a biocompatible polymer [129], however some chemical modifications could modulate its toxicity. A series of studies in vitro by Schipper et al. highlighted the role of the deacetylation degree (DD) in chitosan-induced toxicity. At high DD, chitosan molecular weight and concentration correlate with its toxicity, whereas by lowering chitosan DD, this correlation vanishes [130,131].

In vivo, Rao et al. reported no significant toxic effects of chitosan in acute toxicity tests in mice [132]. No eye or skin irritation in rabbits and Guinea pigs were also respectively verified. In a seminal work, Arai et al. found that the LD50 of chitosan is comparable to sucrose (>16 g/kg) in oral administration to mice [133]. Additionally, exposure of rat nasal mucosa to chitosan solutions at 0.5% (*w*/*v*) over 1 h caused no significant changes in mucosal cell morphology compared to control [134].

Investigation of chitosan capability to regulate innate and adaptive immune responses has been performed by different researchers. For example, it has been shown that chitosan has divergent effects on cytokine production. Lee et al. indicated that diluted chitosan at concentrations of 0.001% and 0.005% increased the expression of IL-2 and IFN-γ in porcine spleen cells. These cytokines have an important role in Th1 lymphocyte proliferation and activation [135]. In contrast, they reported that chitosan had no effect on expression of Th2-related cytokines including IL-4, IL-5, IL-6, and IL-10. However, Wen and colleagues report that chitosan nanoparticles could considerably up-regulate both the mRNA expression of Th1 (IL-2 and IFN-γ) and Th2 (IL-10) cytokines in splenocytes of immunized mice [136]. Even in this case, the nanostructured material stimulates different responses from the molecular form, likely due to shape and size effects. In addition, chitosan has been shown to enhance macrophage activation through toll-like receptor 4 (TLR-4) as stated by Zhang et al. [137]. Some studies reported that chitosan can also increase migration and differentiation of stem cells as well as increased activation of macrophages, leading to potential consequences on immune modulation [138,139].

**Figure 6:**
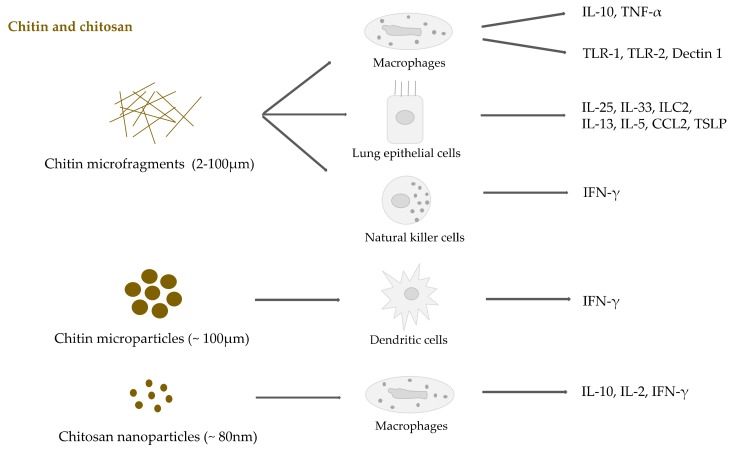
Cytokine release and receptor expression induced by chitin and chitosan NMs.

Although some important results have been already obtained using chitin and chitosan molecules, there are still several aspects of chitin/chitosan-based NMs’ or nanocomposites’ interactions with immune system to be elucidated. Chitin and chitosan nanostructure induced cytokine release by different immune cells and upregulation of membrane receptors is recapitulated in Figure 6.

### 2.4. Seaweed Derived Carrageenan and Alginate

**Figure 7:**
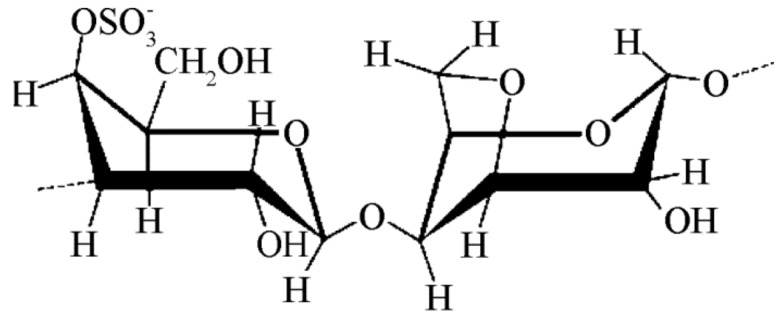
Schematic representation of a κ-carrageenan macromolecule.

Carrageenans belong to a family of high molecular weight sulfated polysaccharides extracted from red seaweeds. The carrageenan macromolecules are formed by alternate units of d-galactose and 3,6-anhydro-galactose (3,6-AG) joined by α-1,3 and β-1,4-glycosidic linkage [140,141] with 15–40% ester-sulfate content (Figure 7). Carrageenan is intensively used in food as a gelling, stabilizing, and thickening agent or as a fat substitute [142]. In addition, its exploitation in the cosmetic, textile, and pharmaceutical industries underlines the commercial importance of this anionic polysaccharide. Unexpectedly, carrageenan shows intrinsic pharmaceutical properties including antitumor, anticoagulant, and anti-hyperlipidemic activity [143,144,145,146].

Carrageenan intake as a food additive is generally safe. Several studies conducted in rats, mice, Guinea pigs, and monkeys demonstrated that carrageenan has very low toxicity and no teratogenicity [147,148,149]. However, other studies have raised concerns about its safety when assembled in nanometric complexes. Catanzaro et al. showed carrageenan induced macrophage cytotoxicity but not lymphocyte cell death [150]. In particular, its administration at high concentrations lead to massive lysosomal storage causing subsequent lysosome rupture and cell death. In a different study, L929 fibroblasts cultured in vitro were not affected by carrageenan/chitosan NPs at concentration from 0.1 to 3 mg/mL [151]. Other authors also observed that high levels of the carrageenan/alginate hydrogels displayed low cytotoxicity, even increasing the polysaccharide concentration at 3.5% [152].

On the other hand, evidence has emerged about carrageenan proinflammatory attitude, probably due to its sulfate groups [153]. Degraded carrageenan (<50 kDa) could lead to a series of immune reactions linked to the activation of NF-κB. Inhibition of THP-1 cell proliferation in vitro, increase of ICAM-1 expression, aggregation of monocytes, and up-regulation of TNF-α expression and secretion have been shown [154]. These events are probably influenced by the molecular weight and the secondary structure of the studied type of carrageenan [155]. The importance of carrageenan concentration for immune response has been highlighted by Yermark and co-researchers, who demonstrated that different types of tested carrageenans up-regulated the expression of IL-6 and TNF-α at high concentration (1µg/mL), while at low concentrations (1–10 ng/mL) their activity was insignificant [156]. Surprisingly, all types of carrageenans also induced the expression of the anti-inflammatory cytokine IL-10 in a dose-dependent manner.

**Figure 8:**
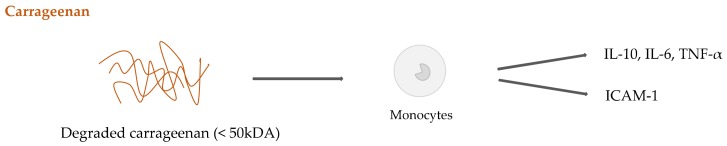
Degraded carrageenan activation of monocytes.

The above mentioned results push further the research to obtain data from standardized nano-complexes with precise size, shape and charge, before their potential wide employment in biomedical applications. An overview of monocyte activation by degraded carrageenan is shown in Figure 8.

**Figure 9:**
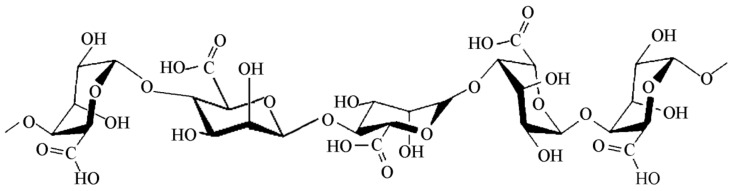
Schematic representation of an alginate macromolecule.

Alginates represent a family of biopolymers formed by alginic acids and its salts derived from brown algae. They consist of linear macromolecules with 1,4-linked β-d-mannuronic acid (M) and 1,4 α-l-guluronic acid (G) residues (Figure 9). Alginate polymer can be arranged in a homogenous (poly-G, poly-M) or heterogenous (M-G) block-like pattern [157]. The most used alginate compositions include alginic acid, sodium alginate, ammonium alginates and calcium alginate. As a carrageenan, alginates are exploited in the food industry as emulsifier, stabilizer, thickener, and texturizer agents, as well in the pharmaceutical industry as a tablet binder, taste masking, thickener, and viscosity increasing agent [158]. There are several biomedical applications explored for alginate-based products including wound healing therapy [159], drug delivery [160,161,162], cell microencapsulation [163], and tissue engineering [164,165].

**Figure 10:**
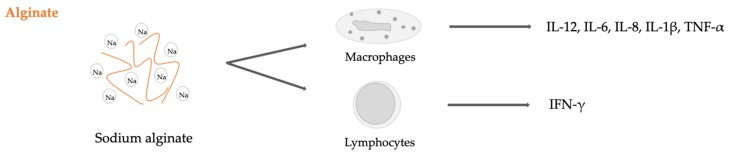
Sodium alginate immune activation of Peripheral Blood Mononuclear Cells (PBMCs).

Like other previously presented polysaccharides, alginate is considered to be a non-toxic and biocompatible material. However, some studies have reported its capability to modulate the immune system, particularly inducing both innate and adaptive responses. Yang et al. showed that alginate causes innate immune response through NF-κB in vitro [166]. Specifically, they tested two concentrations of sodium alginate at 1 mg/mL and 3 mg/mL on a murine macrophage-like cell line, RAW2647, up to 48 h. They found that prior to stimulation, NF-κB was localized in the cytoplasm and after incubation with the two concentrations of alginate solution, NF-κB translocated to the nucleus, in the presence or absence of LPS administration. Furthermore, time and dose-dependent production of pro-inflammatory cytokines such as IL-1β, IL-6, Il-12, and TNF-α was detected. Similar results were reported by Ge et al. analyzing three types of alginate materials characterized by low viscosity, high viscosity and particulate alginate, both in vivo and in vitro. They treated C57BL/6J (B6) mice with alginate and tested peripheral blood using ELISA for cytokine production [167]. The authors showed that alginate materials did not affect the viability of lymphocytes but induced the production of cytokines, such as IL-1β, IL-8, TNF-α and IFN-γ (Figure 10). The treatment with particulate alginate induced very high cytokine expression. They also treated dendritic cells, macrophages, and splenocytes isolated from mice demonstrating the expression of cells surface markers. Low viscosity and particulate alginates were more effective than high viscosity alginates in activating dendritic cells. The secretion of inflammatory cytokines can be also obtained by the release of alginate oligomers (guluronate and mannuronate) as demonstrated by Iwamoto et al. These authors found that enzymatically depolymerized alginate oligomers induced the secretion of inflammatory cytokines from human mononuclear cells [168]. In a different investigation, Yamamoto and colleagues observed that highly purified alginate oligomers of defined structure induced TNF-α secretion from RAW2647 cells in a structure-dependent manner [169].

Because of the large amount of alginate biomedical applications, it will be important to deeply understand nanosized alginate immune-response to improve the existing materials and create new alginate-based drug delivery systems.

## 3. Conclusions

In the present review, we describe the most commercially relevant natural polysaccharides for applications in the biomedical field. We focus on their interaction with the immune system, which represents the major limiting factor of nanosized materials once verified their direct toxicity. In our opinion, the interpretation of data regarding some proinflammatory effects collected in several investigations with diverse experimental models require special attention. Colloidal suspensions of polysaccharide NPs often include fragments of very different size and shape. Since polysaccharide secondary structures play an important role in the interaction with biomolecules and modulate their activity, it would be crucial to standardize protocols of purification, synthesis, and physicochemical characterization of the described NMs in order to improve data reproducibility. Besides single-polysaccharide NMs, nanocomposites also show favorable features and improved material properties. Their immune interactions are limited to the nano-bio interface in the case of films or big fragments with nano-structured surfaces. Differently, polysaccharide composite NPs can be internalized by cells through different routes and activate several intracellular signals. Once inside the cells, nanocomposite NPs can be degraded and their components differentially modulate the transduction pathways, leading to unknown responses with different kinetics. Taking all of the above into consideration, we believe that an interdisciplinary effort to investigate polysaccharide NMs’ interactions with immune system will be beneficial to create novel tools for biomedical applications.

## Figures and Tables

**Table 1:** List of natural polysaccharides classified according to their origin.

Origin	Polysaccharide
Plants	Starch, cellulose, glucomannan, pectin, hemicellulose, gums, mucilage
Algae	Agar, galactans, alginates, carrageenans
Animals	Chitin, chitosan, hyaluronic acid, glycosaminoglycans, cellulose
Bacteria	Dextran, levan, polygalactosamine, gellan, xanthan, cellulose
Fungal	Elsinan, chitin, chitosan, pollulan, yeast glucans

**Table 2:** Diameter, length, and aspect ratio of the different cellulose nano-object.

Cellulose Nano-Object	Diameter (nm)	Length (nm)	Aspect Ratio	References
Cellulose microfibers	10–40	>1000	100–150	[69,70]
Cellulose nanofibers	4–10	~200	50–20	[71]
Cellulose nanowhiskers	2–20	100–600	10–100	[21,72,73]
Bacterial cellulose nanofibers	100	-	-	[62]
Cellulose nanoparticles	50–300	-	-	[74,75]

The symbol “-“ means undefined measure or range.
